# Ultrasound shows swollen joints are the better proxy for synovitis than tender joints in DMARD-naïve early psoriatic arthritis

**DOI:** 10.1093/rap/rkab086

**Published:** 2021-11-15

**Authors:** Sayam R Dubash, Oras A Alabas, Xabier Michelena, Leticia Garcia-Montoya, Gabriele De Marco, Mira Merashli, Richard J Wakefield, Paul Emery, Dennis McGonagle, Ai Lyn Tan, Helena Marzo-Ortega

**Affiliations:** 1 NIHR Leeds Biomedical Research Centre, Leeds Teaching Hospitals NHS Trust; 2 Leeds Institute of Rheumatic and Musculoskeletal Medicine, University of Leeds, Leeds; 3 School of Biological Sciences, Faculty of Biology, Medicine and Health, The University of Manchester, Manchester, UK; 4 Rheumatology Unit, Vall d’Hebron Hospital Universitari, Barcelona, Spain; 5 Division of Rheumatology, Department of Internal Medicine, American University Hospital, Beirut, Lebanon

**Keywords:** PsA, spondyloarthropathies, spondyloarthritis, US, synovitis, physical examination, clinical examination, disease activity

## Abstract

**Objective:**

To evaluate the relationship between clinical examination/US synovitis in DMARD-naïve early PsA.

**Methods:**

Eligible patients underwent matched clinical/US 44-joint assessment for tender and/or swollen joints (TJ/SJ) and US synovitis [grey scale (GS) ≥ 2 or power Doppler (PD) ≥ 1]. Statistical agreement between TJ/SJ, GS ≥ 2 and PD ≥ 1 was calculated by prevalence-adjusted and bias-adjusted κ (PABAK). To derive probabilities of GS ≥ 2/PD ≥ 1, mixed-effects logistic regression-modelled odds of US synovitis in TJ/SJ were conducted.

**Results:**

In 155 patients, 5616 joints underwent clinical/US examination. Of these joints, 1039 of 5616 (18.5%) were tender, 550 of 5616 (9.8%) were swollen, 1144 of 5616 (20.4%) had GS ≥ 2, and 292 of 5616 (5.2%) had PD ≥ 1. GS ≥ 2 was most prevalent in concomitantly tender and swollen joints [205 of 462 (44%)], followed by swollen non-tender joints [32 of 88 (36.4%)], tender non-swollen joints [148 of 577 (25.7%)] and non-tender non-swollen joints (subclinical synovitis) [759 of 4489 (16.9%)]. Agreement between SJ/PD ≥ 1 was high at the individual joint level (82.6–96.3%, PABAK 0.65–0.93) and for total joints combined (89.9%, PABAK 0.80). SJ/GS ≥ 2 agreement was greater than between TJ/GS ≥ 2 [73.5–92.6% *vs* 51.0–87.4% (PABAK 0.47–0.85 *vs* PABAK 0.35–0.75), respectively]. Swelling was independently associated with higher odds of GS ≥ 2 [odds ratio (OR) (95% CI); 4.37 (2.62, 7.29); *P* < 0.001] but not tenderness [OR = 1.33 (0.87, 2.06); *P* = 0.192]. Swelling [OR = 8.78 (3.92, 19.66); *P* < 0.001] or tenderness [OR = 3.38 (1.53, 7.50); *P* = 0.003] was independently associated with higher odds of PD ≥ 1.

**Conclusion:**

Synovitis (GS ≥ 2 and/or PD ≥ 1) was more likely in swollen joints than in tender joints in DMARD-naïve, early PsA. Agreement indicated that swollen joints were the better proxy for synovitis, adding to greater understanding between clinical and US assessments.

Key messages

- This is the largest cross-sectional study evaluating the association between clinical joint examination and US findings in DMARD-naïve early PsA.
- US synovitis is more likely in swollen joints, with better agreement than tender joints.
- Subclinical synovitis (grey scale≥2) remains a significant finding, accounting for 16.9% of concomitantly non-tender non-swollen joints in newly diagnosed early PsA.

## Introduction

Psoriatic Arthritis (PsA) is associated with considerable heterogeneity, including different phenotypes and lack of laboratory biomarkers, which can lead to diagnostic difficulty [1]. The initial diagnosis and assessment of PsA is dependent upon identifying joint swelling and tenderness by clinical examination, a fundamental skill and core outcome in the clinician’s assessment of disease activity. Joint examination findings are not only central to management decisions, but they are crucial elements of inclusion criteria in randomized controlled clinical trials and of eligibility criteria for prescription of biologic drugs in clinical practice [2]. The tender/swollen joint counts (TJC/SJC) are also key components in composite outcome measures such as the PsA response criteria (PsARC), disease activity score 28 (DAS28), composite psoriatic disease activity index (CPDAI), disease activity index for PsA (DAPSA) and PsA disease activity score (PASDAS) and constitute separate domains needed to achieve the PsA treatment targets for minimal disease activity or very low disease activity criteria [3, 4]. Ultimately, persistent joint swelling is associated with progressive joint erosion, pain and functional loss [5, 6]. In common with RA, synovitis in PsA may manifest as swelling of joints on clinical examination. However, PsA patients often report joint pain and may have tender joints without swelling, which may relate to other pathologies, such as enthesitis, the significance of which is not clearly understood.

US is increasingly used in PsA diagnosis and management to identify joint synovitis, and peri-tendon/tendon/entheseal inflammation, owing to its superior sensitivity over clinical examination [7]. However, previous studies have reported a disparity between clinical and US findings, including a high prevalence of subclinical synovitis [8–11]. More recent analyses in established RA patients (treated with DMARDs) have demonstrated an association between clinically swollen joints and US synovitis, which was not found in the context of tender joints [12]. Pathophysiological evidence indicates that PsA differs from RA, with primary enthesopathy followed by secondary synovial inflammation in PsA, in contrast to primary synovitis in RA [13, 14]. In clinical practice, swollen joints and presence of synovitis justify initiation of systemic therapy owing to known responsiveness to biologic DMARDs [15]. Structural damage in PsA is also linked with reduced quality of life and increased risk of death [16]. Although some PsA patients may exhibit minimal disease, others can suffer greater articular inflammation, which needs to be identified and treated. The inhibition of synovitis with DMARDs also plays a key role in halting structural damage in PsA, but how US findings relate to clinical examination is not well understood [17, 18]. Although early US imaging is an excellent confirmatory tool in the diagnosis and management of PsA, not all patients will undergo US in real-world practice, owing to several factors, including lack of resources and time constraints. On clinical examination, visible and palpable articular swelling often negates the need for US, on assumption that it translates to synovitis, but tender joints are more difficult to interpret given their wider association with pathologies. Nonetheless, clinicians frequently face challenging clinical decisions centred on disease activity status based on TJC/SJC. Tenderness may be influenced by non-inflammatory pathologies, such as osteoarthritis and FM, particularly in advanced disease, which can result in disproportionately high TJC [19]. In clinical practice, this is problematic, yet highly relevant. Therefore, understanding the relationship between clinical joint tenderness/swelling and US synovitis remains crucial for improving early identification of disease, decision-making and therapeutic intervention.

The objective of this study was to determine the association between joint clinical examination findings and US synovitis in early PsA. To avoid possible confounders, we chose to explore a cohort of DMARD-naïve early PsA patients.

## Methods

### Patients

In this single-centre cross-sectional prospective observational inception cohort study, 155 consecutive DMARD-naïve PsA patients attending the Leeds Early Arthritis clinic between December 2013 and October 2019 were recruited into the Leeds Spondyloarthropathy Register for Research and Observation (SpARRO). Eligibility was determined by age (≥18 years), ≥3/5 points scored in the classification for PsA criteria (CASPAR), and no previous or current exposure to DMARDs [20]. Ethical approval was granted by the Leeds West Research Ethics Committee (LG03/028), and all patients provided written informed consent in accordance with the Declaration of Helsinki.

### Clinical details and examination

A full clinical history and examination was conducted by the study rheumatologist, unaware of US findings. Examinations of individual joints were recorded as tender or non-tender and swollen or non-swollen, as per TJC/SJC (78/76), and matched for the corresponding 44 US-scanned joints per patient. Clinical enthesitis was assessed via the MASES (13 physical sites of entheseal insertion: Achilles, first and seventh costochondral joints, anterior superior iliac spines, posterior superior iliac spines, iliac crest and fifth lumbar spinous process) and US entheses scanned (for sites, see *Image* *scoring* below) according to the domains of the OMERACT.

### US examination

#### Image acquisition

Examination of 44 joints per patient was conducted using the GE Logiq E9 US machine and linear ML 15–6 MHz or small-footprint linear array 18–8 MHz transducer by trained and experienced sonographers blinded to clinical details, laboratory results and previous imaging. The clinical/US examinations occurred on the same day and followed a protocol-driven procedure standardized according to EULAR guidelines [21]. The wrists (radiocarpal, intercarpal and ulnar-carpal regions), MCP joints 1–5, PIP joints 1–5, DIP joints 2–5, knees (suprapatellar pouch, medial and lateral parapatellar recesses), ankles (tibiotalar) and MTP joints 1–5 were scanned in longitudinal/transverse planes at the dorsal aspect. One of four experienced sonographers, each with >5 years of experience, conducted the US scans. Sonographer calibration was regularly conducted at least twice per year at the same institution to ensure that performance, quality, image interpretation, scoring and recording of results were maintained to a high and consistent standard and in line with the study protocol.

#### Image scoring

Semi-quantitative scoring for grades of grey scale (GS) and power Doppler (PD) were recorded individually for each scanned joint on a scale from zero to three, with the highest GS and PD documented at sites in the wrists and knees. Semi-quantitative GS and PD grades were dichotomized to enable analysis to explore US synovitis. US GS = 0–1 was defined as normal because it is frequently prevalent in healthy controls, whereas GS = 2–3 is more frequently associated with disease [22]. US synovitis was defined as GS ≥ 2 (i.e. GS ≥ 2 + PD ≥ 0) or PD ≥ 1 (i.e. GS ≥ 1 + PD ≥ 1; GS ≥ 2 + PD ≥ 1 was also assessed). Five entheseal sites were assessed via US as part of the modified Glasgow ultrasound enthesitis scoring system (mGUESS), which included the Achilles enthesis, plantar fascia, proximal and distal insertions of the patellar ligament, and the quadriceps tendon insertion into the patella. Hypoechogenicity, thickening, power Doppler, calcifications, enthesophytes and bursitis (except at the quadriceps tendon) were assessed according to OMERACT definitions.

### Statistical analysis

Percentages were used to describe categorical variables, means/medians and SD/interquartile range (IQR) for continuous variables. Baseline clinical and US assessments were analysed at the patient level (TJC/SJC) and the individual joint level. Statistical agreement was calculated between for tender and/or swollen joints (TJ/SJ; individual joint level) independently and US synovitis, dichotomized for GS/PD grades using the prevalence-adjusted and bias-adjusted κ (PABAK). The κ value (PABAK) for agreement was interpreted using a probabilistic benchmarking method: poor = 0.00; slight = 0.01–0.20; fair = 0.21–0.40; moderate = 0.41–0.60; substantial = 0.61–0.80; almost perfect = 0.81–1.00 [23].

Mixed effects logistic regression was used to model the odds of US synovitis in a joint, according to clinical tenderness, swelling and joint type. Each US outcome (GS ≥ 2, PD ≥ 1 or GS ≥ 2 + PD ≥ 1) was modelled separately; predictors were entered simultaneously for each model. Joints (level 1) were nested within patients (level 2) in these models with random intercepts and slopes. Interactions between tenderness and swelling, which allowed the extent to which tenderness predicted the US outcome to vary according to whether swelling was also present, were investigated using likelihood ratio tests. All tests were two tailed; the level of statistical significance was prespecified at 5% (*P* < 0.05), and estimates were derived with 95% CIs.

To reflect the fact that underlying odds of US synovitis differ between sites of joints, another variable was created for the site of joint affected (JSite) for conducting the logistic regression analysis and receiver operating characteristic (ROC). Statistical analyses were performed using Stata v.16.1 (College Station, Texas, StataCorp LLC) and WinPEPI (PEPI-for-Windows) v.11.65.

## Results

### Patients and characteristics

The mean (S.d.) age was 44.4 (12.8) years, and 52.9% were female. The median duration from PsA diagnosis was 1.1 months (IQR 0–3.0) and median symptom duration 12 months (IQR 7–30), indicating an early PsA cohort. An oligoarticular phenotype was most prevalent [99 of 155 (63.9%) patients; polyarticular in 56 of 155 (36.1%)]. Characteristics of the cohort are detailed in Table 1.

**Table 1:** Baseline characteristics of the early DMARD-naïve PsA cohort

Baseline characteristics	*n* = 155
Age, mean (S.d.), years	44.4 (12.8)
Male, *n* (%)	73 (47.1)
Symptom duration, median (IQR), months	12 (7–30)
Time from diagnosis to examination, median (IQR), months	1.1 (0–3.0)
Early morning stiffness, median (IQR) min	60 (15–120)
TJC (78), median (IQR)	7 (3.0–14.0)
SJC (76), median (IQR)	2 (1.0–7.0)
TJC (44), median (IQR)	5 (2–10)
SJC (44), median (IQR)	2 (1–6)
Dactylitis, *n* (%)	69 (44.5)
Current psoriasis, *n* (%)	153 (98.7)
PASI, median (IQR)	2.7 (0.5– 4.6)
Nail dystrophy, *n* (%)	93 (60)
mNAPSI, median (IQR)	0 (0–6)
BMI, median (IQR), kg/m^2^	28.5 (24.6–32.0)
Disease phenotype	
Oligoarthritis, *n* (%)	99 (63.9)
Polyarthritis, *n* (%)	56 (36.1)
DIP joint disease, *n* (%)	17 (11.4)
Axial disease, *n* (%)	22 (14.6)
Arthritis mutilans, *n* (%)	0 (0)
Inflammatory markers	
CRP, median (IQR), mg/l	<5 (<5–14.9)
Elevated (>10 mg/l), *n* (%)	54 (34.8)
Not elevated (≤10 mg/l), *n* (%)	101 (65.2)
ESR, median (IQR), mm/hr	13 (6–26)
Serological markers	
HLA-B27 positive, *n* (%)	15 (12.6)
ANA positive, *n* (%)	3 (2.0)
RF positive, *n* (%)	3 (2.1)
ACPA positive, *n* (%)	8 (5.3)
Patient-reported outcomes	
PsAQoL, median (IQR)	6 (1–12)
DLQI, median (IQR)	3 (0–7)
HAQ, median (IQR)	0.732 (0.25–1.375)

DLQI: dermatology life quality index; HLA-B27: human leucocyte antigen-B27; IQR: interquartile range; mNAPSI: modified nail psoriasis severity index; PASI: psoriasis area severity index; PsAQoL: PsA-specific quality of life.

### Prevalence of clinical and US findings

Of the 5616 joints evaluated, a cumulative total of 1039 of 5616 (18.5%) were clinically tender, 550 of 5616 (9.7%) were clinically swollen, 462 of 5616 (8.2%) were both tender and swollen, and 577 of 5616 (10.3%) were tender in the absence of swelling (tender non-swollen). GS ≥ 2 synovitis was detected in at least one joint in 152 of 155 (98.1%) patients, and PD ≥ 1 in 130 of 155 (83.9%) patients. In total, GS ≥ 1 was present in 2273 of 5616 (40.5%) joints, GS ≥ 2 in 1144 of 5616 (20.4%) joints, PD ≥ 1 in 292 of 5616 (5.2%) joints, and combined GS ≥ 2 + PD ≥ 1 in 162 of 5616 (2.9%) joints. Total GS = 1 was present in 1129 of 5616 (20.1%), whereas GS = 1 + PD ≥ 1 was observed in only 50 of 5616 (0.89%) joints.

In concomitant tender and swollen joints, GS ≥ 2 synovitis was proportionally greatest [205 of 462 (44%)], followed by swollen joints without tenderness [32 of 88 (36.4%)], tender joints without swelling [148 of 577 (25.7%)] and non-tender non-swollen joints [759 of 4489 (16.9%)] as shown in Table 2. Likewise, for detection of PD ≥ 1, the same order was followed, albeit at lower proportions (26.2, 18.2, 6.1 and 2.7%, respectively). A disproportionate number of US-scanned joints did not have clinical swelling or tenderness [4489 of 5616 (79.9%)], and of all GS ≥ 2 synovitis detected, subclinical synovitis was present in more than half of all joints [759 of 1144 (66.3%)], compared with tender or swollen joints, respectively [TJ: 353 of 1144 (30.9%); SJ: 237 of 1144 (20.7%)]. Of all joints with PD ≥ 1 synovitis, 172 of 292 (58.9%) had either clinical tenderness or swelling (or both); interestingly, 120 of 292 (41.1%) had subclinical PD ≥ 1 synovitis. GS = 1 synovitis was similar between categories for subclinical *vs* swollen *vs* tender joints, respectively [891 of 4489 (19.8%) *vs* 107 of 550 (19.5%) *vs* 215 of 1039 (20.7%)]. The frequencies of individual US GS/PD grades in tender/swollen joints are shown in Supplementary Table S1, available at *Rheumatology Advances in Practice* online.

**Table 2:** Frequencies of tender and swollen joints for US synovitis

Tender/swollen joint combinations	GS ≥ 2	PD ≥ 1	GS ≥ 2 and PD = 0	GS ≥ 2 and PD ≥ 1
Total tender (*n* = 1039 of 5616)	353; 34.0% (6.2%)	156; 15.1% (2.8%)	216; 20.8% (3.8%)	137; 13.2% (2.4%)
Total swollen (*n* = 550 of 5616)	237; 43.1% (4.2%)	137; 24.9% (2.4%)	115; 20.9% (2.0%)	122; 22.2% (2.2%)
Both tender and swollen (*n* = 462 of 5616)	205; 44.4% (3.7%)	121; 26.2% (2.2%)	98; 21.2% (1.7%)	107; 23.2% (1.9%)
Tender and not swollen (*n* = 577 of 5616)	148; 25.7% (2.6%)	35; 6.1% (0.6%)	118; 20.5% (2.1%)	30; 5.2% (0.5%)
Swollen and not tender (*n* = 88 of 5616)	32; 36.4% (0.6%)	16; 18.2% (0.3%)	17; 19.3% (0.3%)	15; 17.1% (0.3%)
Neither tender nor swollen (*n* = 4489 of 5616)	759; 16.9% (13.5%)	120; 2.7% (2.1%)	666; 14.8% (11.9%)	93; 2.1% (1.7%)

Values are given as a frequency and percentage of the category-specific clinical combination and the percentage of the total patient cohort (in parentheses).

GS: grey scale; PD: power Doppler.

The joint-specific prevalence of TJ, SJ, GS and PD grades is outlined in Supplementary Table S2, available at *Rheumatology Advances in Practice* online. In the feet, GS ≥ 2 was frequently detected, in 495 of 1034 (47.9%) joints. The most prevalent site of GS ≥ 2 was at the MTP1 (46.5%; also a frequently observed site for OA), followed by MTP2–4 (range 37.5–51.7%) and wrists (30.1%). Power Doppler (PD ≥ 1) was most prevalent at the wrists (17.5%) and MTP1 (12.6%).

### Agreement analysis

#### Individual joint level, split by joint type

**Fig. 1:**
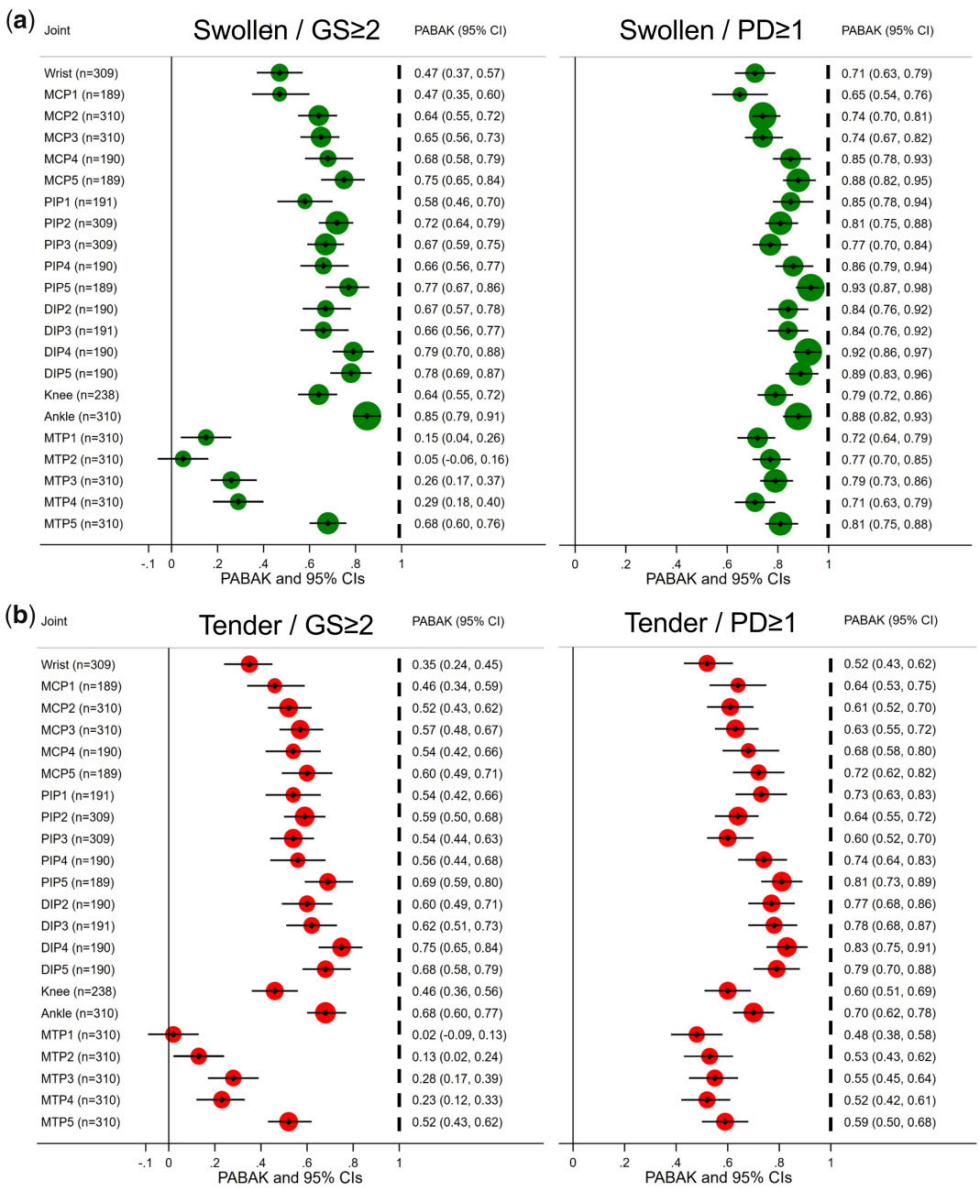
Forest plots of agreement between tender or swollen joints and US synovitis per joint site (**A**) Overall statistical agreement between swollen joints and PD ≥ 1 was highest, and for GS ≥ 2 it remained high for the majority of joints except in the feet (poor at MTP1–4; good at MTP5). (**B**) Overall moderate/high agreement is shown between tender joints and GS ≥ 2/PD ≥ 1 in the hands. In the feet, lower agreement was found, mirroring the pattern found in swollen joints. GS: grey scale; PABAK: prevalence-adjusted and bias-adjusted κ; PD: power Doppler.

Agreement at the individual joint level was highest between SJ/PD ≥ 1 synovitis with the highest agreement (82.6–96.3%, PABAK 0.65–0.93), closely followed by GS ≥ 2 (except feet), as illustrated in Fig. 1A. Agreement between TJ/PD ≥ 1 synovitis was high, but lower than that observed for SJ (72.9–91.1%, PABAK 0.46–0.82), as shown in Fig. 1A and B. It is noteworthy that in tender joints GS ≥ 2 was found in 353 of 1039 (34%), and in non-tender joints GS ≥ 2 occurred in 791 of 4577 (17.3%), whereas in swollen joints GS ≥ 2 was detected in 237 of 550 (43.1%), and in non-swollen joints GS ≥ 2 occurred in 907 of 5066 (17.9%). Statistical agreement was much lower in the feet [e.g. SJ/GS ≥ 2 at MTP1–4 (53.3–64.5%, PABAK 0.07–0.29)], with the exception of MTP5, where it remained high (GS: 83.9%, PABAK 0.68; PD: 90.7%, PABAK 0.81). The same pattern was mirrored for TJ/GS ≥ 2, with less overall agreement than SJ/GS ≥ 2 [TJ/GS ≥ 2: 51.0–87.4% agreement, PABAK 0.35–0.75; SJ/GS ≥ 2: 73.5–92.6%, PABAK 0.47–0.85 (excluding MTP1–4 for TJ/SJ)].

#### Joint level, combining all joints

Combining all joints, statistical agreement was higher for SJ/US synovitis (GS ≥ 2 or PD ≥ 1) than for TJ [SJ/PD ≥ 1: 89.9% (89.1–90.7), PABAK 0.80 (0.78–0.81); SJ/GS ≥ 2: 78.3% (77.2–79.4), PABAK 0.57 (0.54–0.59); TJ/PD ≥ 1: 81.9% (80.9–82.9), PABAK 0.64 (0.62–0.66); TJ/GS ≥ 2: 73.7% (72.6–74.9), PABAK 0.47 (0.45–0.50); Table 3]. Percentage negative agreement (Pneg) was higher than positive agreement (Ppos) given that the sample included a greater proportion of non-tender/non-swollen and GS < 2 (Table 3). Greater positive agreement was present for joint tenderness if also swollen [GS ≥ 2: 58.7% (PABAK −0.05); PD ≥ 1: 40.4% (PABAK −0.30)] than for joint tenderness if not swollen [GS ≥ 2: 19.9% (PABAK 0.53); PD ≥ 1: 9.6% (PABAK 0.74)]. To understand the interplay between tenderness, swelling and US findings, we proceeded to model synovitis as a function of tenderness and swelling simultaneously by logistic regression analysis.

**Table 3:** Percentage category-specific agreement for combinations of tender/swollen joints and US synovitis

All joints	GS (2–3 *vs* 0–1; 1144 of 5616)				PD (1–3 *vs* 0; 292 of 5616)				GS ≥ 2 + PD ≥ 1 *vs* GS 0–1 and/or PD 0 (245 of 5616)			
	Overall agreement (95% CI)	Pneg (95% CI)	Ppos (95% CI)	PABAK (95% CI)	Overall agreement (95% CI)	Pneg (95% CI)	Ppos (95% CI)	PABAK (95% CI)	Overall agreement (95% CI)	Pneg (95% CI)	Ppos (95% CI)	PABAK (95% CI)
Tender (1039 of 5616)	73.7% (72.6, 74.9)	83.7% (82.8, 84.5)	32.3% (29.8, 34.9)	0.47 (0.45, 0.50)	81.9% (80.9, 82.9)	89.7% (89.1, 90.3)	23.4% (20.4, 26.6)	0.64 (0.62, 0.66)	82.0% (81.0, 83.0)	89.8% (89.2, 90.5)	21.3% (18.2, 24.3)	0.64 (0.62, 0.66)
Swollen (550 of 5616)	78.3% (77.2, 79.4)	87.2% (86.5, 87.9)	28.0% (25.2, 30.8)	0.57 (0.54, 0.59)	89.9% (89.1, 90.7)	94.5% (94.1, 95.0)	32.5% (28.5, 36.6)	0.80 (0.78, 0.81)	90.2% (89.4, 91.0)	94.7% (94.3, 95.1)	30.7% (26.5, 34.8)	0.80 (0.79, 0.82)
Tender and/or swollen (1127 of 5616)	73.3% (72.1, 74.4)	83.2% (82.4, 84.1)	33.9% (31.3, 36.4)	0.47 (0.44, 0.49)	80.9% (79.8, 81.9)	89.0% (88.4, 89.7)	24.2% (21.3, 27.3)	0.62 (0.60, 0.64)	81.0% (80.0, 82.0)	89.2% (88.5, 89.8)	22.2% (19.5, 25.2)	0.62 (0.60, 0.64)
Tender and swollen (462 of 5616)	78.7% (77.6, 79.8)	87.6% (86.3, 88.3)	25.5% (22.7, 28.3)	0.57 (0.55, 0.60)	90.1% (90.1, 91.6)	95.1% (94.7, 95.5)	32.1% (27.9, 36.4)	0.82 (0.80, 0.83)	91.2% (90.5, 92.0)	95.3% (94.9, 95.7)	30.3% (25.9, 34.8)	0.82 (0.81, 0.84)
Tender if swollen (462 of 550)	47.5% (43.3, 51.6)	27.9% (22.3, 33.8)	58.7% (54.2, 62.9)	−0.05 (−0.13, 0.03)	35.0% (31.1, 39.1)	28.7% (23.7, 34.1)	40.4% (35.5, 45.2)	−0.30 (−0.38, −0.22)	32.7% (28.8, 36.7)	28.3% (23.2, 33.5)	36.6% (31.7, 41.6)	−0.35 (−0.42, −0.27)
Tender if not swollen (577 of 5066)	76.6% (75.4, 77.8)	86.3% (85.5, 87.0)	19.9% (17.4, 22.7)	0.53 (0.51, 0.55)	86.9% (86.0, 88.0)	93.0% (92.4, 93.5)	9.6% (6.8, 12.7)	0.74 (0.72, 0.76)	87.4% (86.5, 88.3)	93.2% (92.6, 93.7)	8.6% (6.1, 11.7)	0.75 (0.73, 0.77)

Values are the percentage overall agreement (%), category-specific proportions of positive (Ppos) and negative (Pneg) agreement and adjusted κ (PABAK) with 95% CIs for tender/swollen joints and US synovitis (all joints combined).

GS: grey scale; PD: power Doppler.

### Clinical examination of entheses and US enthesitis

Compared with 71 of 155 (45.8%) patients with clinical enthesitis (MASES≥1), US enthesopathy (mGUESS) was present in 133 of 155 (85.8%) patients [median (IQR): 3 (1–6)]. However, no relevant statistical associations were found between mGUESS and SJC, TJC or MASES (negative binomial regression; *P* > 0.05).

#### Logistic regression analysis

Preliminary modelling in 5616 joints from 155 patients offered no evidence that the difference in the odds of US synovitis associated with joint swelling varied according to whether a joint was also tender (GS ≥ 2: *P* = 0.404; PD ≥ 1: *P* = 0.463; GS ≥ 2 + PD ≥ 1: *P* = 0.744). Interaction terms were removed from the final models.

**Fig. 2:**
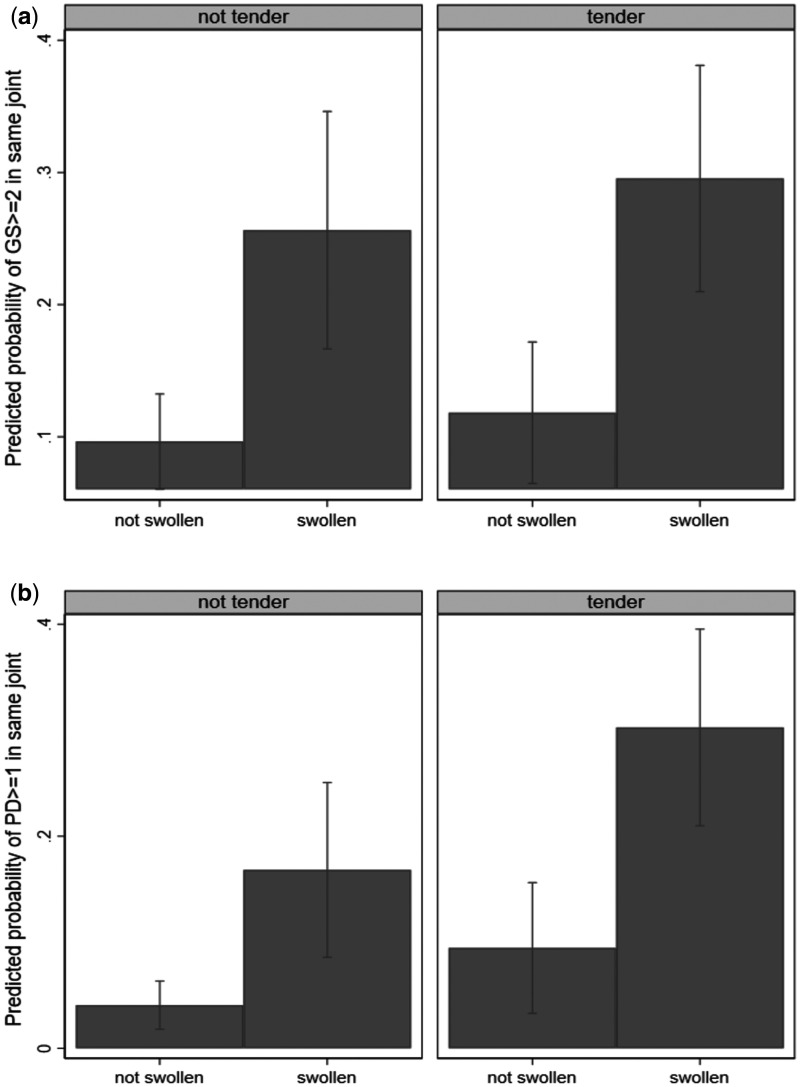
Predicted probabilities of grey scale ≥ 2 and power Doppler ≥ 1 being present according to presence of tenderness and/or swelling Estimations are for MCP2 for illustration. (**A**) Swollen joints were associated with a greater probability of GS ≥ 2 synovitis, but tender joints were not. (**B**) Joint swelling or tenderness was independently associated with higher odds of PD ≥ 1 synovitis. GS: grey scale; PD: power Doppler.

In the average patient, swelling was associated with higher odds of there being GS ≥ 2 in a joint [odds ratio (OR) = 4.37 (95% CI 2.62, 7.29), *P* < 0.001]; however, in the presence or absence of swelling, tenderness was not associated with an additional increase in the odds of GS ≥ 2 being present [OR = 1.33 (0.87, 2.06), *P* = 0.192; Fig. 2A]. Independently, joint swelling [OR = 8.78 (3.92, 19.66), *P* < 0.001] and tenderness [OR = 3.38 (1.53, 7.50), *P* = 0.003] were associated with higher odds of PD ≥ 1 (Fig. 2B). Similar results were obtained for concomitant GS ≥ 2 + PD ≥ 1 [swelling: OR = 8.21 (3.24, 20.81), *P* < 0.001; tenderness: OR = 3.66 (1.41, 9.46), *P* = 0.008].

**Fig. 3:**
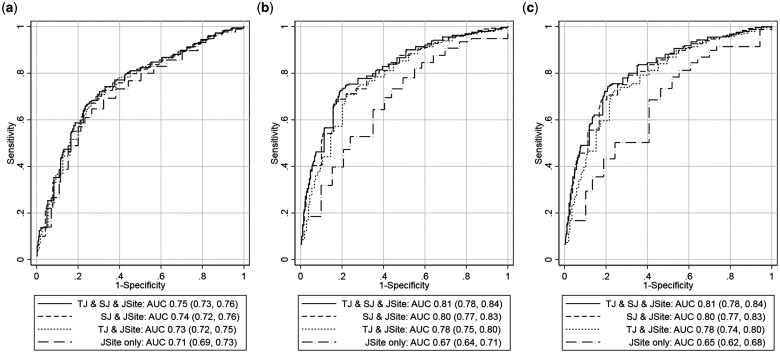
Receiver operating characteristic curve models of US synovitis for joint tenderness and/or swelling Receiver operating characteristic curves for fixed predictions from models of GS ≥ 2 (**A**), PD ≥ 1 (**B**) and GS ≥ 2 + PD ≥ 1 (**C**) at the joint level, including different combinations of the predictors tenderness (TJ), swelling (SJ) and joint site (JSite). The graph plots show the true-positive rate (sensitivity) against the false-positive rate (1 − specificity = 1 − true negative), illustrating the diagnostic ability of clinical examination (TJ, SJ or TJ + SJ) in detecting US synovitis. The site of joint affected (JSite) influenced the model for each US synovitis parameter and was therefore included in the analysis as a variable. GS: grey scale; PD: power Doppler.

An ROC model produced very marginal differences between the area under the curve (AUC) for TJ, SJ and TJ + SJ. The predictive value of adding TJ to SJ and vice versa was an additional 0.01 for TJ, and 0.02–0.03 for SJ, for TJ + SJ + JSite, the highest AUC achieved for all US synovitis categories. Despite being associated with the US outcomes that included PD independently of SJ, tenderness did not add substantively to the prediction of each outcome over and above swelling alone (Fig. 3).

## Discussion

This is the largest cross-sectional study, to our knowledge, to explore clinical joint findings in relationship to US synovitis in a DMARD-naïve early PsA cohort. The results from this study confirmed that SJ were associated with a greater probability of having US synovitis (GS ≥ 2 or PD ≥ 1) than TJ. A greater association was found between SJ/US synovitis over TJ/US synovitis, which was previously reported in other settings [8, 12]. Statistical agreement was generally high for clinical TJ and/or SJ and US synovitis (GS ≥ 2 or PD ≥ 1) at individual joints, and overall SJ were the better proxy. However, the lower positive agreement compared with higher negative agreement indicated that the presence of clinically tender or swollen joints was still lacking compared with the known higher sensitivity of US for synovitis.

Proportionally, GS ≥ 2 synovitis was most prevalent in swollen joints (43.1%), followed by tender joints (34.0%) and then non-tender non-swollen (subclinical) joints (16.9%). Joints that were concomitantly tender and swollen (TJ + SJ) attained low percentages of positive agreement comparable to SJ/US synovitis (GS ≥ 2 or PD ≥ 1). Positive agreement was higher for TJ in the presence of swelling, whereas TJ in the absence of swelling (tender non-swollen joints) rendered lower agreements (GS ≥ 2: 58.7% *vs* 19.9%; PD ≥ 1: 40.4% *vs* 9.6%), indicating a weaker association. A disproportionate number of joints were clinically unaffected (non-tender and non-swollen) [*n* = 4489 of 5616 (79.9%)] compared with clinically affected joints (tender, swollen or both) [*n* = 1589 of 5616 (20.1%)], with two-thirds of all detected GS ≥ 2 synovitis [759 of 1144 (66.3%)] seen in subclinical joints. Nonetheless, subclinical synovitis (16.9%) was similar to that shown in other studies, although its relevance for prognostication is unknown [10, 11, 24]. Our results are consistent with the known reduced sensitivity of clinical examination and the disparity reported for clinical and US outcomes [8].

In GS ≥ 2 synovitis, the presence of PD rather than GS was more strongly associated with swollen than tender joints. In comparison to joint swelling, tenderness was not considered to increase the odds of GS ≥ 2 [OR: 4.37 (SJ) *vs* 1.33 (TJ)]. However, the probability of detecting PD synovitis (PD ≥ 1 or GS ≥ 2 + PD ≥ 1) was increased for tender joints and for swollen joints independently [OR: 8.78, 8.21 (SJ) *vs* 3.38, 3.66 (TJ), respectively]. Thus, the association seemingly appeared to be driven by SJ more than TJ, and US synovitis by PD established stronger associations with SJ/TJ than GS. The ROC curve analysis confirmed that there was little difference to the odds of a swollen joint having GS ≥ 2 synovitis when joint tenderness was added. Only marginal differences existed between TJ, SJ and TJ + SJ for each US synovitis category, and the ROC AUC did not alter substantially when TJ was added to SJ (AUC improved by 0.01) for each US synovitis outcome either.

Results from the present study are in agreement with previous reports in other settings showing stronger association between SJ and US synovitis over TJ in early and established RA [12, 25]. Moderate correlation was shown between clinically SJ and US (GS/PD) synovitis compared with TJ (weak/not significant) in PsA at the patient level, but no association was found with extra-capsular disease [8]. In a longitudinal study of 47 PsA patients, an association between PD and SJC, CRP, ESR and DAS28, was reported, but not for TJC or the DAPSA [9].

Synovitis was determined by GS ≥ 2; however, in certain joints GS = 1 may be relevant, and there remains uncertainty over what represents physiological *vs* pathological synovitis for GS = 1 grade, including whether it is PsA- or non-PsA-related change. Indeed, the EULAR–Outcome MEasures in Rheumatology (EULAR-OMERACT) consensus-based scoring system recognizes GS ≥ 2 or PD ≥ 1 in the definition for grading synovitis [26]. This guidance also includes low-grade GS without PD (GS 1–2) detectable in healthy individuals, despite the known propensity at specific joint sites that may also be affected by OA (e.g. MTP1) [22].

These study results confirmed a higher prevalence of synovitis, particularly subclinical GS, in the feet (47.9%) than hands, which might be explained by the greater degree of biomechanical stress to which weight-bearing joints are subjected [27]. This resulted in paradoxically lower statistical agreement at small joints of the feet (MTP5 excluded). Whether low-grade GS resembles healthy physiology or early pathological findings can be indistinguishable, particularly in the absence of clinical findings, but regression of GS following treatment suggests that some might represent active disease [28].

Nonetheless, examination of joints is not without subjectivity. Tender joints are common in PsA, but may be misjudged as swollen. Obesity, for example, is highly prevalent in PsA (37%) and skin psoriasis (29%) compared with RA (27%) or the general population (18%), making clinical assessments for synovitis more difficult [29]. Distinguishing the underlying cause of tenderness, for example from enthesitis, synovitis or FM, can also pose challenges. To mitigate the effect of disproportionately high tender count, the swollen and tender joint count ratio (STR) and tender–swollen joint count difference (TSJD) have been developed, but these outcomes may not reflect underlying pathology [30, 31].

The lack of association between US enthesitis and clinically examined sites (mGUESS and TJC, SJC and MASES) might be reflective of differences in outcomes or the mismatch between tenderness and extra-synovial pathologies. These results raise important questions on the pathological representation of TJ/SJ beyond synovitis, including the association with PsA-related microanatomical enthesitis, tendinopathy/peri-tendon inflammation in early PsA. Other studies have shown a mixture of results, with or without correlation between clinical and US findings. Tenderness in acute dactylitis is associated with flexor tenosynovitis more often than synovitis, and chronicity corresponds to the ‘cold’ non-tender form [32]. At large entheses (patellar tendon origins and Achilles enthesis), clinical and US enthesitis have recently shown an association [33]. In contrast, tenosynovitis and peri-tendinitis had very low concordance between clinical and US findings [34].

Limitations of this study include the concomitant use of NSAIDs, either intermittently or regularly (81 of 155 patients), which might have affected low levels of inflammation on US, given that US GS/PD could be masked by NSAIDs [35]. The majority of patients, however, had no exposure to CSs; only 3 of 155 within 6 weeks of their assessment. This study focused on the assessment of synovitis given the recognized link from persistent joint inflammation to bone erosions leading to progressive structural and functional damage, yet a limitation might arguably be the entheseal outcomes used for extra-synovial pathologies [36]. Given that tender non-swollen joints are common in PsA, and only one-quarter had GS ≥ 2 synovitis (25.7%), one consideration to investigate the cause of tenderness further would be to use enhanced imaging techniques, namely high-resolution MRI of small joints for assessment of digital microanatomical enthesitis, including flexor pulleys [37]. These study findings have important implications for the basis of treatment decisions. Finally, these findings support the use of US for early PsA diagnosis, especially in the presence of tender non-swollen joints. Further research on tender joints might improve the understanding of pathologies in early PsA. Conducting US is not possible at every patient consultation; therefore, US could be prioritized for those with predominantly tender joints to confirm early PsA.

### Conclusions

Swollen joints were the better proxy for US synovitis (GS ≥ 2 and/or PD ≥ 1) compared with tender joints in DMARD-naïve early PsA. Overall agreement was lower for MTP1–4 examination and US synovitis (GS ≥ 2 or PD ≥ 1), confirming a disparity and indicating the added sensitivity of US assessment in the feet. Synovitis (GS ≥ 2 and/or PD ≥ 1) was more likely in swollen joints, whereas tenderness was not associated with significant increase in the odds of GS ≥ 2 but did increase the odds of PD ≥ 1 in a joint. These findings add to the understanding of how clinical examination relates to underlying pathologies on US imaging, which might help to improve the management of early PsA.

## Supplementary Material

rkab086_Supplementary_Data
