# Supplementary material for: Ultrasound shows swollen joints are the better proxy for synovitis than tender joints in DMARD-naïve early psoriatic arthritis
Source: Rheumatol Adv Pract. 2021 Nov 15;5(3):rkab086. doi: 10.1093/rap/rkab086 (PMC8908782; doi:10.1093/rap/rkab086)
Supplement: rkab086_Supplementary_Data [file rkab086_Supplementary_Data.docx]

**Supplementary Files:**

Supplementary Table S1. Prevalence of tender joints, swollen joints, ultrasound GS/PD synovitis per grade per joint.

| **Joint** | **Tender** | **Swollen** | **GS=0** | **GS=1** | **GS=2** | **GS=3** | **PD=0** | **PD=1** | **PD=2** | **PD=3** |
| --- | --- | --- | --- | --- | --- | --- | --- | --- | --- | --- |
| Wrist (n=309) | 70 (22.7%) | 35 (11.3%) | 95 (30.7) | 121 (39.2) | 84 (27.2) | 9 (2.9) | 246 (82.0) | 31 (10.3) | 23 (7.7) | 0 |
| MCP1 (n=190) | 42 (22.1%) | 37 (11.0%) | 105 (55.3) | 40 (21.1) | 33 (17.4) | 12 (6.3) | 174 (91.6) | 9 (4.7) | 6 (3.2) | 1 (0.5) |
| MCP2 (n=310) | 67 (21.6%) | 49 (15.8%) | 161 (51.9) | 112 (36.5) | 27 (8.7) | 10 (3.2) | 288 (92.9) | 9 (2.9) | 9 (2.9) | 4 (1.3) |
| MCP3 (n=310) | 72 (23.2%) | 51 (16.5%) | 177 (57.1) | 93 (30.0) | 28 (9.0) | 12 (3.9) | 287 (92.6) | 7 (2.3) | 11 (3.6) | 5 (1.6) |
| MCP4 (n=190) | 35 (18.4%) | 17 (9.0%) | 106 (55.8) | 61 (32.1) | 14 (7.4) | 9 (4.7) | 179 (94.2) | 8 (4.2) | 2 (1.1) | 1 (0.5) |
| MCP5 (n=190) | 24 (12.6%) | 8 (4.2%) | 124 (65.3) | 46 (24.2) | 14 (7.4) | 6 (3.2) | 183 (96.3) | 4 (2.1) | 2 (1.1) | 1 (0.5) |
| PIP1 (n=190) | 27 (14.2%) | 15 (7.9%) | 123 (64.7) | 24 (12.6) | 39 (20.5) | 4 (2.1) | 179 (98.4) | 2 (1.1) | 1 (0.6) | 0 |
| PIP2 (n=309) | 64 (20.7%) | 35 (11.3%) | 253 (81.9) | 25 (8.1) | 21 (6.8) | 10 (3.2) | 285 (96.0) | 4 (1.4) | 6 (2.0) | 2 (0.7) |
| PIP3 (n=310) | 66 (21.3%) | 39 (12.6%) | 250 (80.7) | 26 (8.4) | 23 (7.4) | 11 (3.6) | 289 (97.0) | 5 (1.7) | 3 (1.0) | 1 (0.3) |
| PIP4 (n=190) | 29 (15.3%) | 13 (6.8%) | 154 (81.1) | 13 (6.8) | 17 (9.0) | 6 (3.2) | 176 (97.8) | 2 (1.1) | 2 (1.1) | 0 |
| PIP5 (n=190) | 19 (10.0%) | 8 (4.2%) | 159 (83.7) | 13 (6.8) | 16 (8.4) | 2 (1.1) | 177 (98.3) | 1 (0.6) | 2 (1.1) | 0 |
| DIP2 (n=190) | 21 (11.1%) | 14 (7.4%) | 160 (84.2) | 11 (5.8) | 17 (9.0) | 2 (1.1) | 187 (98.4) | 1 (0.5) | 1 (0.5) | 1 (0.5) |
| DIP3 (n=190) | 20 (10.5%) | 12 (6.3%) | 151 (79.8) | 19 10.0) | 19 (10.0) | 1 (0.5) | 187 (98.4) | 1 (0.5) | 2 (1.1) | 0 |
| DIP4 (n=190) | 15 (7.9%) | 7 (3.7%) | 157 (82.6) | 18 (9.5) | 13 (6.8) | 2 (1.1) | 187 (98.4) | 1 (0.5) | 2 (1.1) | 0 |
| DIP5 (n=190) | 20 (10.5%) | 10 (5.3%) | 166 (87.4) | 12 (6.3) | 11 (5.8) | 1 (0.5) | 186 (97.9) | 2 (1.1) | 2 (1.1) | 0 |
| Knee (n=308) | 59 (19.2%) | 34 (11.0%) | 192 (62.3) | 64 (20.8) | 42 (13.6) | 10 (3.3) | 299 (97.1) | 4 (1.3) | 5 (1.6) | 0 |
| Ankle (n=310) | 47 (15.2%) | 21 (6.8%) | 263 (84.8) | 33 (10.7) | 9 (2.9) | 5 (1.6) | 306 (98.7) | 4 (1.3) | 0 | 0 |
| MTP1 (n=310) | 64 (20.7%) | 19 (6.1%) | 70 (22.6) | 96 (31.0) | 104 (33.6) | 40 (12.9) | 271 (87.4) | 24 (7.7) | 13 (4.2) | 2 (0.7) |
| MTP2 (n=310) | 69 (22.3%) | 31 (10.0%) | 68 (21.9) | 82 (26.5) | 140 (45.2) | 20 (6.5) | 296 (95.5) | 11 (3.6) | 3 (1.0) | 0 |
| MTP3 (n=310) | 71 (22.9%) | 33 (10.7%) | 89 (28.7) | 82 (26.5) | 122 (39.4) | 17 (5.5) | 295 (95.2) | 7 (2.3) | 8 (2.6) | 0 |
| MTP4 (n=310) | 74 (23.9%) | 42 (13.6%) | 119 (38.4) | 75 (24.2) | 100 (32.3) | 16 (5.2) | 293 (94.5) | 9 (2.9) | 7 (2.3) | 1 (0.3) |
| MTP5 (n=310) | 64 (20.7%) | 20 (6.5%) | 201 (64.8) | 63 (20.3) | 40 (12.9) | 6 (1.9) | 293 (94.5) | 9 (2.9) | 6 (1.9) | 2 (0.7) |

GS: grey scale; PD: power Doppler

Supplementary Table S2. Ultrasound GS/PD synovitis per grade in combinations of tender and swollen joints.

| **All joints** | **GS=0** | **GS=1** | **GS=2** | **GS=3** | **PD=0** | **PD=1** | **PD=2** | **PD=3** |
| --- | --- | --- | --- | --- | --- | --- | --- | --- |
| All tender (n=1039/5616) | 471 (45.3%) | 215 (20.7%) | 255 (24.5%) | 98 (9.4%) | 883 (85.0%) | 63 (6.1%) | 75 (7.2%) | 18 (1.7%) |
| All swollen (n=550/5616) | 206 (37.5%) | 107 (19.5%) | 154 (28.0%) | 83 (15.1%) | 413 (75.1%) | 53 (9.6%) | 69 (12.6%) | 15 (2.7%) |
| Both tender and swollen (n=462/5616) | 173 (37.5%) | 84 (18.2%) | 133 (28.8%) | 72 (15.6%) | 341 (73.8%) | 46 (10.0%) | 61 (13.2%) | 14 (3.1%) |
| Tender and not swollen (n=577/5616) | 298 (51.7%) | 131 (22.7%) | 122 (21.1%) | 26 (4.5%) | 542 (93.9%) | 17 (3.0%) | 14 (2.4%) | 4 (0.7%) |
| Swollen and not Tender (n=88/5616) | 33 (37.5%) | 23 (26.1%) | 21 (23.9%) | 11 (12.5%) | 72 (81.8%) | 7 (8.0%) | 8 (9.1%) | 1 (1.1%) |
| Neither tender nor swollen (n=4489/5616) | 2839 (63.2%) | 891 (19.9%) | 657 (14.6%) | 102 (2.3%) | 4369 (97.3%) | 85 (1.9%) | 31 (0.7%) | 4 (0.1%) |

GS: grey scale; PD: power Doppler
